# Spin‐Selective Oxygen Evolution in Chiral Molecule‐Intercalated Layered Double Hydroxides

**DOI:** 10.1002/advs.76090

**Published:** 2026-06-16

**Authors:** Chih‐Ying Huang, Cheng‐Rong Wu, Yang‐Sheng Lu, Yu‐Ying Chang, Chia‐Che Chang, Tsung‐Hsin Liu, Che‐Lun Lee, Jessie Shiue, Yu‐Chang Lin, Wei‐Tsung Chuang, Huang‐Ming Tsai, Ya‐Lun Ho, Jau‐Wern Chiou, Hua‐Shu Hsu, Shao‐Sian Li, Chia‐Chun Chen, Way‐Faung Pong, Chun‐Wei Chen

**Affiliations:** ^1^ International Graduate Program of Molecular Science and Technology (NTU‐MST) National Taiwan University Taipei Taiwan; ^2^ Molecular Science and Technology Program Taiwan International Graduate Program (TIGP) Academia Sinica Taipei Taiwan; ^3^ Department of Materials Science and Engineering National Taiwan University Taipei Taiwan; ^4^ Department of Materials Science and Mineral Resources Engineering National Taipei University of Technology Taipei Taiwan; ^5^ Department of Applied Physics National Pingtung University Pingtung County Taiwan; ^6^ Graduate Institute of Nanomedicine and Medical Engineering College of Biomedical Engineering and International Ph.D. Program in Biomedical Engineering College of Biomedical Engineering Taipei Medical University Taipei Taiwan; ^7^ Institute of Atomic and Molecular Sciences Academia Sinica Taipei Taiwan; ^8^ National Synchrotron Radiation Research Center Hsinchu Taiwan; ^9^ Research Center for Electronic and Optical Materials National Institute for Materials Science (NIMS) Ibaraki Japan; ^10^ Department of Applied Physics National University of Kaohsiung Kaohsiung Taiwan; ^11^ Department of Chemistry National Taiwan Normal University Taipei Taiwan; ^12^ Department of Physics Tamkang University New Taipei Taiwan; ^13^ Center for Condensed Matter Sciences and Center of Atomic Initiative for New Materials (AI‐MAT) National Taiwan University (NTU) Taipei Taiwan

**Keywords:** chiral‐induced spin selectivity, in situ observation, layered double hydroxides, molecule intercalation, oxygen evolution reaction

## Abstract

The chiral‐induced spin selectivity (CISS) effect offers a novel paradigm for designing high‐performance catalysts for spin‐dependent oxygen evolution reactions (OER). Layered double hydroxides (LDHs), are widely used for oxygen evolution reaction (OER) due to their superior electrocatalytic activity and stability in alkaline environments. Here, we demonstrate that intercalating chiral phenylalanine molecules into CoFe‐LDH induces spin‐polarized OER via the CISS effect, while simultaneously expanding the interlayer spacing. The resulting chiral–inorganic hybrid interface directs the reaction along a lower‐energy pathway, promoting the formation of triplet O_2_, and exhibits outstanding OER performance with a lower overpotential of 245 mV at 10 mA cm^−2^, as well as faster charge‐transfer kinetics compared to its achiral counterpart. Using in situ XANES, in situ Raman spectroscopy, and nanoscale scanning electrochemical cell microscopy (SECCM), we further uncover the fundamental origin of this chiral‐induced spin‐selective behavior. This study establishes a general strategy for designing advanced, stable oxide‐based electrocatalysts, where intercalated chiral molecules manipulate spin dynamics to improve reaction kinetics and selectivity.

## Graphical abstract

This hybrid electrocatalyst utilizes chiral phenylalanine intercalation within layered double hydroxides to boost oxygen evolution. Acting as a spin filter via the chiral‐induced spin selectivity effect, the material directs electron transfer along a lower‐energy pathway. This process accelerates triplet oxygen formation, significantly reducing overpotentials and improving kinetic efficiency for stable, high‐performance water splitting.

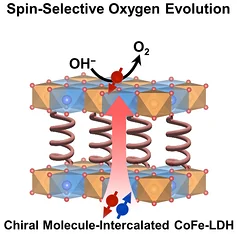

## 1 Introduction

Electrochemical water splitting is a promising strategy for sustainable hydrogen production; however, its efficiency is hindered by the kinetically sluggish oxygen evolution reaction (OER) [1]. The OER involves complex four‐electron transfer kinetics and a spin‐restricted process, wherein singlet‐state reactants (H_2_O or OH^−^) must be converted into triplet‐state O_2_, posing a major bottleneck to the overall reaction efficiency [2]. This change in electron spin multiplicity is a spin‐forbidden process according to quantum mechanical selection rules and therefore requires additional energy to proceed, contributing to the high overpotential [3, 4]. The resulting spin mismatch increases the formation energy barrier for the OER and can also lead to the generation of undesirable byproducts such as hydrogen peroxide (H_2_O_2_), [5, 6] ultimately reducing the overall catalytic efficiency.

Various strategies for directly manipulating electron spin at the catalyst–electrolyte interface have been proposed to promote spin‐allowed pathways and enhance OER performance [7, 8]. One approach involves applying an external magnetic field to ferromagnetic catalysts to promote parallel spin alignment in the reaction intermediates [9, 10], thereby making the oxygen evolution pathway more energetically favorable. However, this method is mainly effective for catalysts exhibiting ferromagnetic (FM) properties, while such a spin‐magnetic effect is absent in non‐FM catalysts such as antiferromagnetic or paramagnetic materials [9]. An alternative strategy is to employ catalysts with intrinsic local spin polarization to enhance the spin‐selected electron transfer efficiency [11, 12]. In such materials, exchange interactions generate localized spin‐selective channels that filter electrons with the appropriate spin orientation required for the OER, without the need for long‐range ferromagnetic ordering [13]. Furthermore, recent advances have revealed that electron transport through chiral molecule‐modified catalysts can generate intrinsic spin polarization even in the absence of an external magnetic field [14]. This phenomenon, known as the chiral‐induced spin selectivity (CISS) effect, is a quantum‐mechanical process in which chiral molecules preferentially transmit electrons with a specific spin orientation [15, 16]. By harnessing the CISS effect, chiral catalysts can produce spin‐polarized electrons, enabling spin‐selective charge transfer and facilitating the formation of triplet‐state O_2_ during the OER [5, 17, 18]. This effect arises from an imbalance between spin‐up and spin‐down electrons upon transmission through chiral molecules, which originates from spin–orbit coupling in non‐centrosymmetric systems [19, 20], leading to a pronounced spin‐dependent transmission probability.

Surface functionalization of metal oxide catalysts with chiral ligands, such as CoO_x_, [5] Fe_3_O_4_ [21, 22], and CuO [6], has been demonstrated to enhance both OER activity and spin selectivity. In contrast to these surface‐functionalized metal oxides, recent studies have introduced the intercalation of chiral molecules within the interlayers of two‐dimensional (2D) transition metal dichalcogenides (TMDs), such as MoS_2_ and TiS_2_ [23, 24, 25, 26]. These chiral molecule–intercalated 2D TMD catalysts exhibit remarkable spin‐selective charge transport owing to strong spin polarization induced by highly oriented chiral molecules confined within the 2D interlayers, leading to significant improvements in spin‐selective OER performance [23, 24]. Nevertheless, the primary challenge of using TMD catalysts for the OER lies in their long‐term stability, especially under alkaline conditions, where they can undergo electrochemical oxidation to form soluble metal ions or unstable metal hydroxides and oxyhydroxides [27]. By contrast, oxide‐based catalysts, such as metal oxides or hydroxides [28, 29], demonstrate prominent stability under alkaline conditions, making them more suitable for robust OER performance in water splitting. Inspired by these advances, we propose utilizing a molecular intercalation strategy applied to layered double hydroxides (LDHs)—a versatile family of two‐dimensional metal hydroxides with tunable compositions, abundant active sites, and excellent stability in alkaline environments [30, 31, 32]. By introducing chiral organic molecules intercalated into the interlayer of CoFe‐LDH, a hybrid chiral–inorganic interface capable of generating and transmitting spin‐polarized electrons during OER is established. We systematically synthesized achiral/chiral molecular‐intercalated CoFe‐LDH using phenylalanine (DL‐, D‐, and L‐forms) and characterized its structural, electronic, and catalytic properties. Chiral LDH exhibits outstanding OER performance compared to achiral LDH, delivering a lower overpotential and faster reaction kinetics. This enhancement arises from the synergistic interplay between molecular intercalation and the CISS effect, which together improve OER efficiency and selectivity. Molecular intercalation increases the LDH interlayer spacing, enhancing active‐site exposure and overall surface area, while the CISS effect imparts intrinsic spin‐filtering capability, enabling spin‐selective electron transport during the anodic OER. By simultaneously enhancing active‐site accessibility and spin‐selective charge transport, molecularly intercalated chiral LDHs lower kinetic barriers and accelerate OER, resulting in exceptional electrocatalytic performance. Moreover, operando spectroscopic techniques are employed to elucidate how chirality‐induced spin polarization modulates spin‐selective charge transfer and governs spin alignment during O─O bond formation in the OER.

Through the combination of in situ x‐ray absorption spectroscopy and Raman spectroscopy, we directly elucidate the relationship between electronic‐structure evolution and molecular chirality in chiral LDH catalysts under operating conditions. In situ XANES identifies the catalytically active sites in CoFe‐LDH, while in situ Raman spectroscopy reveals the formation of reactive oxygen intermediates during OER. Together with electrochemical analysis, these operando measurements provide compelling evidence for spin‐dependent reaction pathways, showing that chiral intercalation modulates the spin‐selectivity of key intermediates, lowers kinetic barriers for O─O bond formation, and accelerates O_2_‐evolution kinetics. Finally, nanoscale scanning electrochemical cell microscopy (SECCM) on individual CoFe‐LDH microplates further unravels chirality‐dependent, spin‐polarized OER behavior at the single‐flake level, corroborating the operando spectroscopic results. These results demonstrate that molecular chirality enhances local catalytic activity via spin‐selective charge transfer. Elucidating these mechanisms is crucial for advancing chirality‐induced spin‐polarized OER catalysis and provides a fundamental framework for the rational design of next‐generation chiral electrocatalysts with improved activity and long‐term stability for sustainable energy conversion.

## 2 Results and Discussion

**FIGURE 1:**
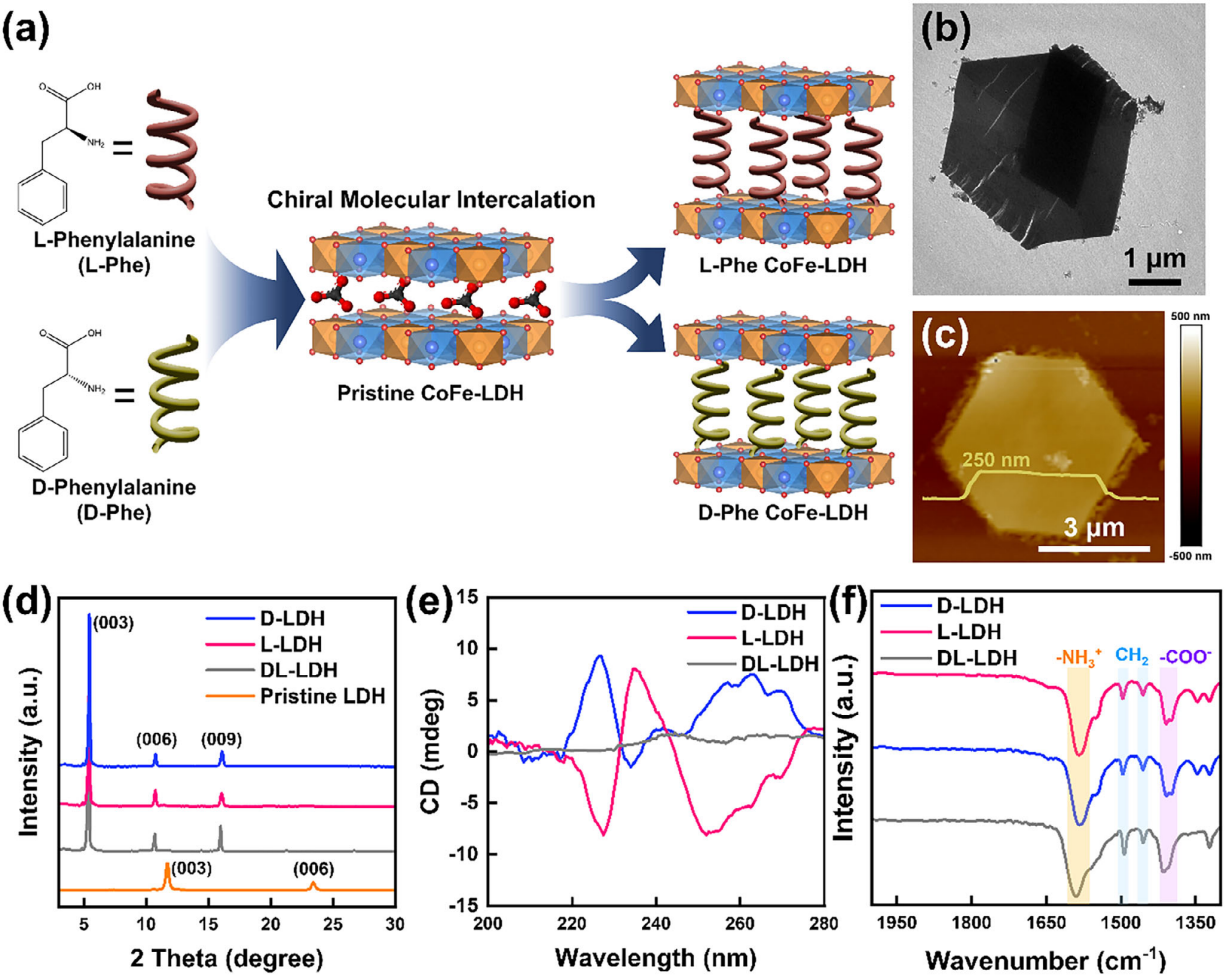
(a) Scheme diagram of chiral molecular intercalation from pristine CoFe‐LDH to chiral CoFe‐LDH. (b) TEM image and (c) AFM image of DL‐LDH. (d) XRD patterns of pristine, DL‐, D‐, and L‐LDH. (e) CD spectra and (f) FTIR spectra of DL‐, D‐, and L‐LDH.

Chiral molecule‐intercalated CoFe‐LDH microplates were synthesized via a co‐precipitation method, where DL‐, D‐, and L‐form phenylalanine (Phe) were used as intercalated chiral molecules to replace the interlayer carbonate (CO_3_^2−^). Figure 1a illustrates the schematic diagram of chiral molecular intercalation, with detailed synthesis method described in the Supporting Information. Interlayer CO_3_^2−^ anions in CoFe‐LDH were replaced by phenylalanine via molecular intercalation, yielding DL‐LDH (racemic Phe), D‐LDH, and L‐LDH. The morphologies of the pristine LDH and Phe‐intercalated LDHs were examined by transmission electron microscopy (TEM), as shown in Figure 1b and Figure S1. Pristine LDHs consist of relatively small, overlapping nanosheets with irregular edges that form micro‐sized aggregates (Figure **S1**). In contrast, Phe‐LDHs display well‐defined layered microplates with sharp edges and substantially larger lateral dimensions (∼5 µm). Their thickness increases to ∼250 nm for D‐, L‐, and DL‐LDH, compared to ∼100 nm for pristine LDH (Figures S2 and S3), consistent with the intercalation of larger Phe molecules into the CoFe‐LDH interlayers. Elemental composition and distribution were characterized by energy‐dispersive X‐ray spectroscopy (EDS). The EDS mapping images reveal a homogeneous distribution of Co and Fe in pristine, DL‐, D‐, and L‐LDH catalysts (Figure S4). X‐ray diffraction (XRD) patterns (Figure 1d) of pristine and Phe‐intercalated LDHs exhibit well‐defined (003) and (006) signatures, confirming the layered structure. Notably, the (003) peaks of DL‐, D‐, and L‐LDH shift toward lower 2θ angles, indicating a pronounced expansion of the interlayer spacing induced by the intercalation of DL‐, D‐, and L‐Phe molecules. The d‐spacing of the (003) plane increases from 0.76 nm in pristine LDH to 1.64 nm in Phe‐LDHs, consistent with the replacement of CO_3_^2−^ anions by larger Phe molecules [33, 34]. The chiroptical properties of the Phe‐LDH materials were further investigated using transmission circular dichroism (CD) spectroscopy. The CD spectra of both D‐ and L‐chiral Phe molecules exhibit a perfect mirror‐symmetric signal below 220 nm, while the DL‐Phe molecule shows no measurable CD response (Figure S5). While the pristine chiral molecules exhibit CD signals only below 220 nm, the D‐ and L‐LDH display pronounced and symmetric CD spectra extending from 225 to 280 nm. In contrast, the DL‐LDH shows no measurable CD response. The CD signals of the L‐and D‐LDH films are notably red‐shifted relative to those of the corresponding L‐Phe and D‐Phe molecules. Notably, a distinct Cotton effect appears at 232 and 243 nm in the chiral LDH thin films, characterized by an inversion of the CD signal across the zero baseline. This spectral shift and the emergence of the Cotton effect are attributed to the splitting of energy levels induced by the intercalation of the chiral Phe molecules into the LDH layers [18, 23]. Therefore, the CD signals in the L‐ and D‐LDH films arise not solely from the chiral Phe molecules but from the hybrid chiral system, reflecting effective chiral transfer. This comprehensive CD analysis provides strong evidence of the distinct chiral properties and directly confirms robust chiral imprinting onto the LDH catalyst's electronic density of states (DOS) [35]. The composition of Phe‐LDHs was further examined by Fourier‐transform infrared (FTIR) spectroscopy. The DL‐, D‐, and L‐LDH catalysts exhibit highly similar FTIR spectra, confirming the presence of analogous chemical functional groups across the three chiral derivatives (Figure 1f). Key vibrational features include a broad band at 1584 cm^−1^, assigned to symmetric NH_3_^+^ stretching and asymmetric COO^−^ stretching, bands at 1495 and 1455 cm^−1^ corresponding to CH_2_ scissoring, and a peak at 1410 cm^−1^ attributable to symmetric COO^−^ stretching. This consistent evidence supports the successful intercalation of Phe molecules into the LDH framework [36, 37].

**FIGURE 2:**
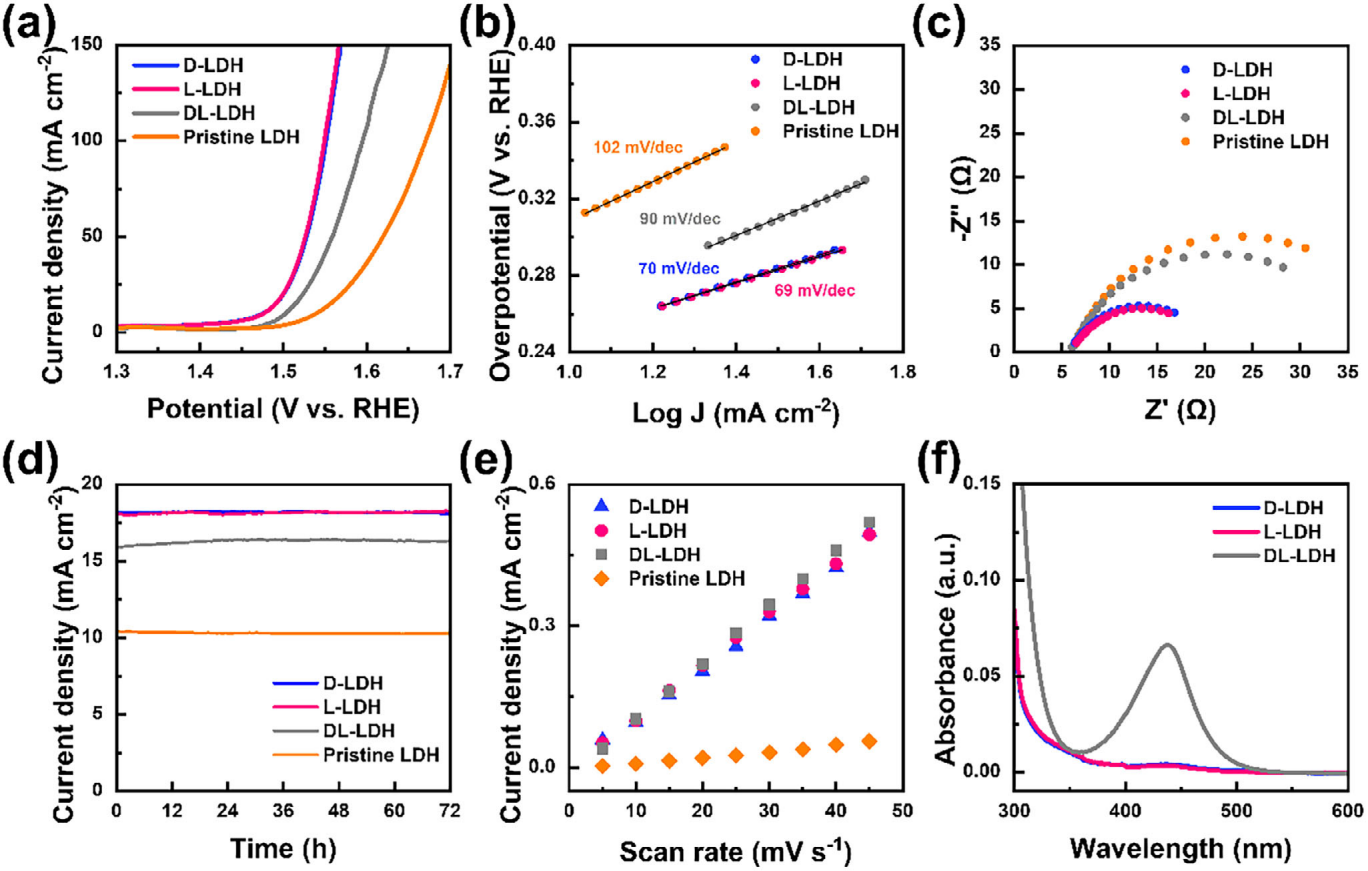
(a) LSV curves, (b) Tafel slopes, (c) EIS spectra, (d) OER stability test, and (e) electrochemical double‐layer capacity (C_dl_) of pristine, DL‐, D‐, and L‐LDH. (f) UV–vis absorption spectra for H_2_O_2_ detection after electrocatalytic OER in neutral electrolyte (0.1 M Na_2_SO_4_).

Electrochemical measurements were conducted using a standard three‐electrode setup, with a Pt wire as the counter electrode and Hg/HgO (1 M KOH) as the reference. Working electrodes were fabricated by drop‐casting catalyst inks of pristine CoFe‐LDH, DL‐LDH, D‐LDH, and L‐LDH onto nickel foam; detailed preparation procedures are provided in the **SI**. The OER performance was evaluated via LSV curves with iR compensation as shown in Figure 2a. The results indicate that the intercalation of Phe molecules noticeably enhances catalytic activity, as evidenced by the lower overpotential of DL‐LDH (270 mV) compared to that of pristine CoFe‐LDH (310 mV) at a current density of 10 mA cm^−2^. Particularly, the D‐LDH and L‐LDH catalysts exhibit the best performance with an overpotential of only 245 mV, which is substantially lower than that of the racemic DL‐LDH. The reaction kinetics were further evaluated via Tafel plots as shown in Figure 2b, where the D‐LDH and L‐LDH catalysts exhibit superior performance with the lowest Tafel slopes of 70 and 69 mV dec^−1^, respectively. This is followed by the racemic DL‐LDH (90 mV dec^−1^), while the pristine CoFe‐LDH shows the highest slope of 102 mV dec^−1^. Electrochemical impedance spectroscopy (EIS) analysis further reveals charge transfer resistances of 35.5, 30.8, 15.1, and 14.7 Ω for pristine, DL‐, D‐, and L‐LDH, respectively, indicating enhanced interfacial charge transfer for the chiral catalysts (Figure 2c). The lower Tafel slopes and charge transfer resistances of D‐ and L‐CoFe LDHs demonstrate that chiral intercalation notably enhances electrocatalytic efficiency and facilitates faster charge transfer compared to their pristine counterparts. Molecularly intercalated chiral LDHs exhibit enhanced OER activity compared to previously reported chiral molecular‐coated or intercalated catalysts (Table S1). The as‐prepared catalysts also exhibit excellent electrochemical stability, retaining their activity after 72 h of continuous operation at 1.5 V vs. RHE (Figure 2d). Such robust stability under alkaline conditions is characteristic of oxide‐based catalysts, making them well‐suited for durable OER performance in water splitting.

To further understand how intercalated DL‐, D‐, and L‐Phe molecules enhance OER performance, the electrochemical surface areas (ECSA) were determined by measuring the double‐layer capacitance (C_dl_) from the non‐Faradaic region of cyclic voltammetry (CV) curves at various scan rates (Figure S6). As shown in Figure 2e, the calculated C_dl_ values for pristine, DL‐, D‐, and L‐LDH are 0.7, 6.0, 5.4, and 5.5 mF cm^−2^, respectively. The substantial improvement in OER performance from pristine LDH to Phe‐LDHs is primarily attributed to the larger Phe anions intercalated into the LDH structure, which expand the interlayer spacing and increase the active surface area [38, 39]. Notably, the chiral D‐ and L‐LDHs exhibit slightly lower C_dl_ values than the achiral DL‐LDH, indicating that the excellent OER performance of chiral LDHs compared to their achiral counterpart arises not from differences in electrochemically active surface area (ECSA) but from intrinsic chirality‐induced spin‐selective effects. In alkaline environments, water oxidation proceeds through the formation of *OH intermediates via both the four‐electron and two‐electron pathways [40, 41]. From a spin perspective, the products of these pathways—O_2_ and H_2_O_2_—exhibit distinct spin states, corresponding to the triplet and singlet configurations, respectively. The production of H_2_O_2_ by DL‐, D‐, and L‐LDH catalysts during OER was investigated using UV–vis absorption spectroscopy with o‐tolidine as a redox probe. Oxidation of o‐tolidine by H_2_O_2_ generates a characteristic absorption peak near 440 nm, enabling direct quantification of the singlet byproduct [42, 43]. As shown in Figure 2f, the achiral DL‐LDH exhibits a pronounced absorption peak around 440 nm, whereas this peak is nearly absent for the chiral D‐ and L‐LDHs. This indicates that the enhanced OER performance of chiral CoFe‐LDH catalysts arises from the suppression of the two‐electron pathway producing H_2_O_2_, thereby favoring the four‐electron pathway leading to triplet O_2_. This distinction highlights the role of chiral LDH as an effective spin filter for OER compared to achiral LDH. In achiral LDH, the reaction pathway lacks spin selectivity, allowing both spin‐up and spin‐down intermediates to form without spin polarization, which results in the generation of both singlet H_2_O_2_ and triplet O_2_. In contrast, chiral LDH preferentially transfers spin‐polarized electrons from OH^−^ ions, producing intermediates (O^−^) with aligned spins and ultimately yielding triplet O_2_, thereby enhancing OER performance. A schematic of spin‐selective electron transfer in achiral vs. chiral LDH is shown in Figure S7.

**FIGURE 3:**
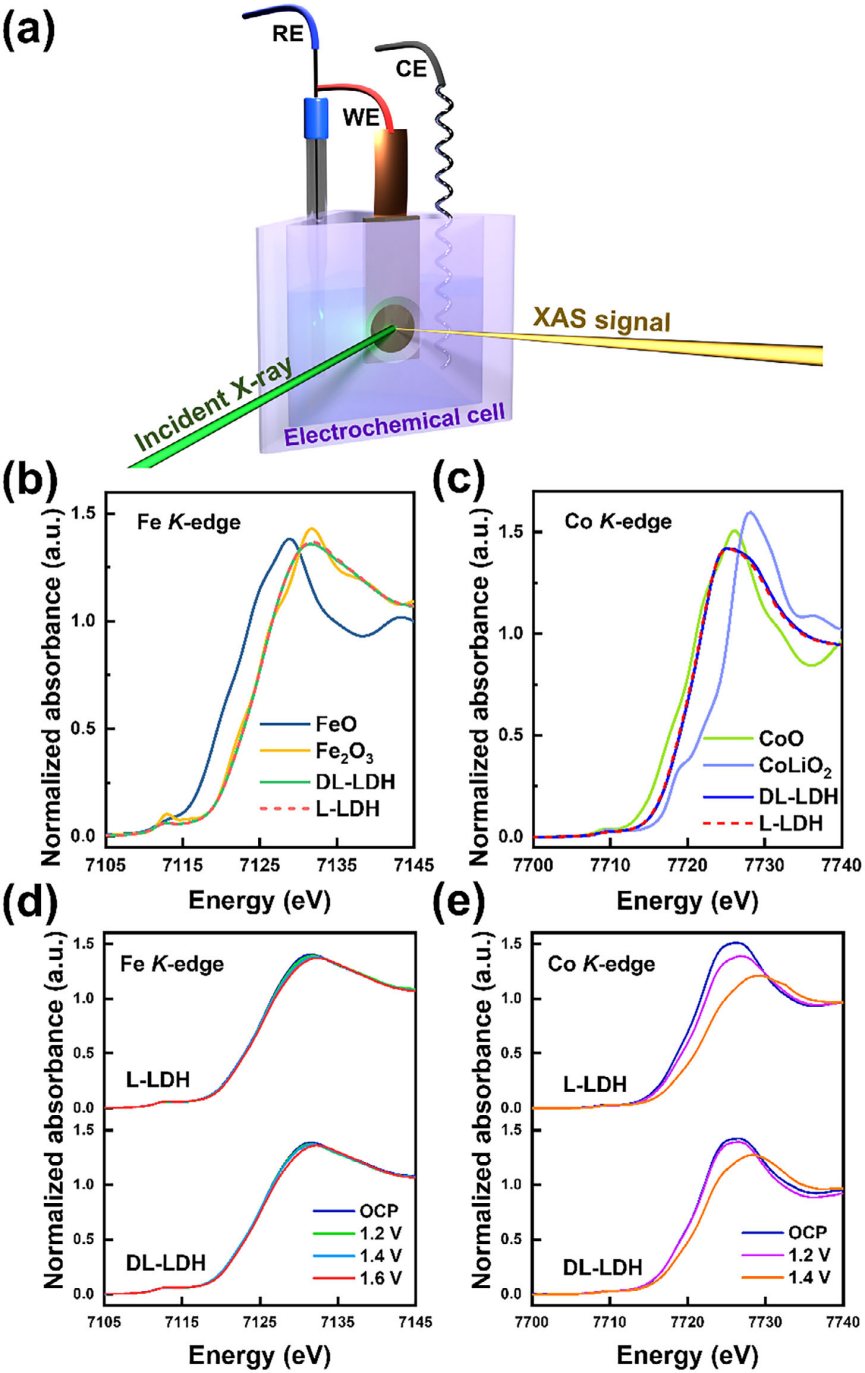
(a) Illustration of in situ XANES setup. The reference electrode, counter electrode, and working electrode were abbreviated to RE, CE, and WE, respectively. The Hg/HgO (1 M KOH) is used as RE; Pt wire is used as CE. The sample is placed by 45° with respect to the incident x‐ray to better face the fluorescence detector. (b) Fe K‐edge XANES of DL‐, L‐LDH compared to FeO and Fe_2_O_3_. (c) Co K‐edge XANES of DL‐, L‐LDH compared to CoO and CoLiO_2_. (d) In situ Fe K‐edge and (e) Co K‐edge XANES of DL‐, and L‐LDH.

To elucidate the fundamental mechanisms underlying chiral‐enhanced OER, we analyzed the evolution of the electronic structures of DL‐ and L‐LDH using in situ X‐ray absorption near‐edge spectroscopy (XANES) at the Co and Fe K‐edges. The experimental configuration for these measurements utilized a three‐electrode system as illustrated in Figure 3a and Figure S8. XANES spectral features are highly sensitive to the oxidation state, spin configuration, and local coordination environment of transition‐metal centers [44]. Initial analysis of the Fe species confirms a predominant Fe^3+^ valence state for both DL‐ and L‐LDH catalysts, with K‐edge positions consistent with the Fe_2_O_3_ standard. This observation is further supported by Fe L_3,2_‐edge soft XAS (sXAS) measurements, which confirm a high‐spin octahedral configuration (Figure 3b and Figure S8). Similarly, the Co species in the as‐prepared catalysts resemble the CoO reference, existing primarily as high‐spin Co^2+^ in an octahedral coordination environment (Figure 3c and Figure S9). To further investigate the OER enhancement of Phe‐LDH catalysts by chiral‐induced spin selectivity, we performed in situ Fe and Co K‐edge XANES spectra in Figure 3d,e during OER. During the OER process, the Fe K‐edge spectra for both DL‐ and L‐LDH remain essentially unchanged, exhibiting negligible energy shifts. The incorporation of Fe into the LDH lattice is known to stabilize the formation of surface oxygen vacancies, which are essential for efficient dioxygen release in synergistic systems [45]. In contrast, the Co K‐edge spectra display pronounced potential‐dependent shifts toward higher energies, indicating that cobalt ions act as the primary active sites and undergo oxidation to higher‐valent states in both catalysts [46, 47, 48]. The edge shift for DL‐LDH occurs at 1.4 V vs. RHE, whereas L‐LDH shows a pronounced shift at 1.2 V vs. RHE. Chirality‐induced spin selectivity enables cobalt ion oxidation in chiral LDH at a lower potential than in achiral LDH, consistent with its superior macroscopic OER performance. In situ XANES provides direct evidence elucidating the interplay between atomic and electronic structures and chirality‐driven enhancement of OER activity in CoFe‐LDH.

**FIGURE 4:**
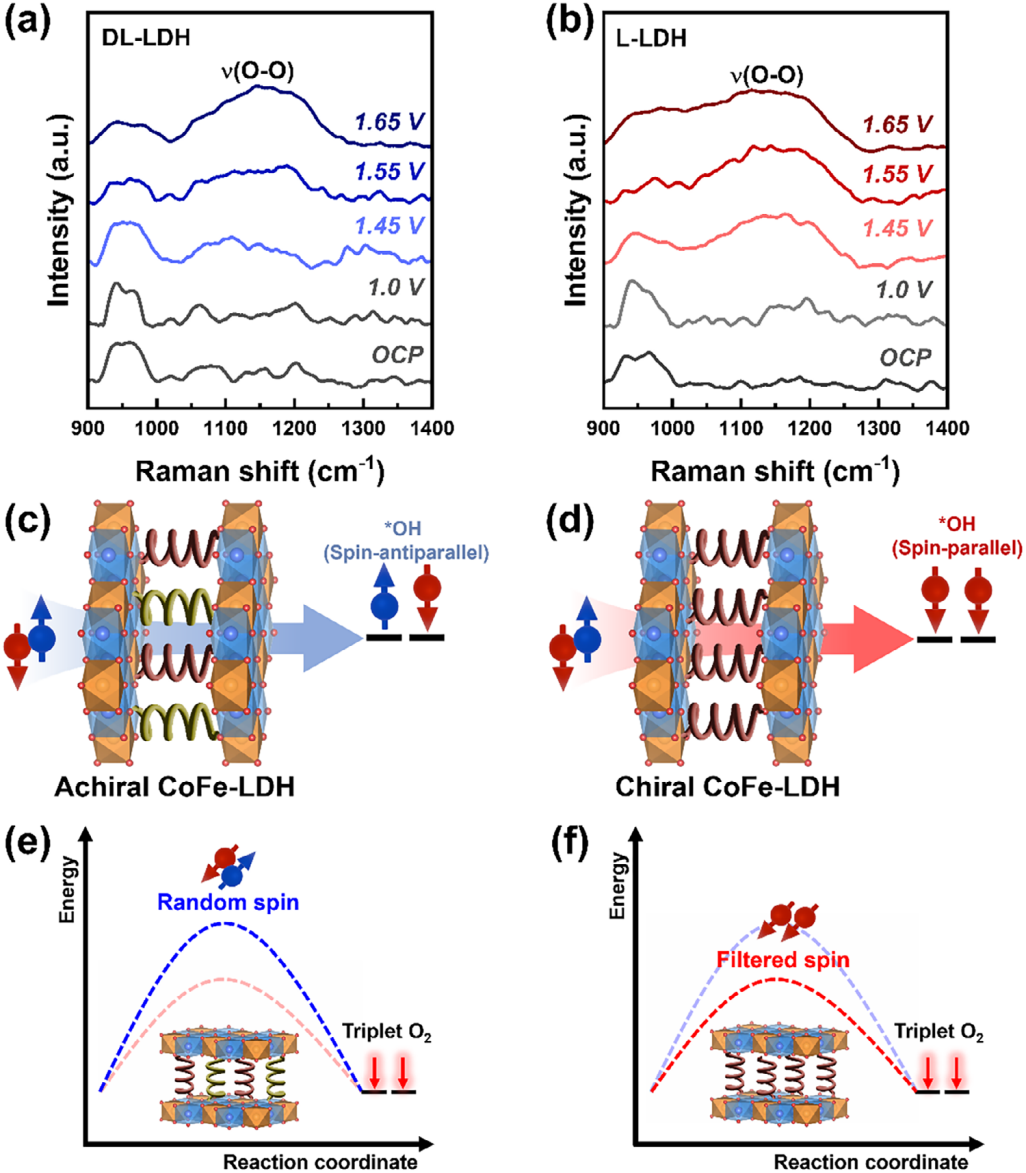
(a) In situ Raman spectra of DL‐, and (b) L‐LDH. (c) Illustration of spin selectivity in achiral LDH and (d) chiral LDH. Schematic energy diagrams for the OER process. (e) Achiral LDH with random spin intermediates showing a higher activation energy barrier. (f) Chiral LDH exhibiting a reduced activation energy barrier due to spin‐polarized intermediates.

To further elucidate the dynamic evolution of reaction intermediates, in situ Raman spectroscopy was performed on both achiral and chiral LDH catalysts under operational OER conditions. This technique is particularly efficacious for identifying distinct vibrational modes associated with metal–oxygen bonding, thereby providing critical insights into the underlying catalytic mechanisms [49]. Figure 4a,b show in situ Raman spectra of DL‐LDH and L‐LDH collected from the open‐circuit potential (OCP) to 1.65 V vs. RHE. Upon entering the OER regime, a broad band in the 1000–1300 cm^−^^1^ region appears, corresponding to the O─O stretching vibration of surface‐bound peroxo intermediates [50]. The band intensity increases with applied potential, reflecting the progressive accumulation of O–O intermediates during catalysis. In particular, the onset potential for direct formation of O─O bond is markedly reduced for the chiral L‐LDH, with the signal emerging at 1.45 V vs. RHE, compared to 1.55 V for the achiral DL‐LDH. This earlier appearance of O─O intermediates directly correlates with the accelerated OER kinetics of L‐LDH relative to DL‐LDH, consistent with the enhanced OER activity observed electrochemically and supporting a chirality‐induced modulation of the reaction pathway.

Moreover, the Raman peaks at 450 and 520 cm^−1^ are assigned to Co─O vibrational modes, indicating that Co(OH)_2_ constitutes the primary component of the CoFe‐LDH host layers (Figure S10). This observation confirms that the intrinsic Co(OH)_2_ structure is well preserved within the bimetallic LDH architecture, consistent with the characteristic vibrational signatures of Co‐based layered double hydroxides [51, 52, 53]. For the DL‐LDH sample, as the applied potential increases to 1.55 V, the Raman peak at 450 cm^−1^ shifts toward lower wavenumbers, indicating the formation of surface metal oxyhydroxide (CoOOH) species [52, 54]. The emergence of surface CoOOH is widely recognized as the active center for electrocatalytic OER [55]. By contrast, L‐LDH undergoes this structural transformation at a lower potential of 1.45 V. This peak shift signifies a transition in the cobalt oxidation state from Co^2+^ to Co^3+^, corresponding to the conversion of Co(OH)_2_ into CoOOH, and is consistent with the in situ XANES results discussed previously [53]. By combining the insights from in situ XANES and Raman spectroscopy, the proposed OER mechanism in chiral molecular–intercalated LDHs can be described as follows. First, according to the lattice oxygen mechanism (LOM) [56, 57], OH^−^ reactants preferentially adsorb onto Co ions, which act as the active sites, consistent with the energy shifts observed in the in situ XANES spectra. Subsequently, Co─OH bond formation occurs in conjunction with a bridging lattice oxygen, leading to the generation of an intermediate species that is detectable by in situ Raman spectroscopy. The corresponding LOM pathway is schematically illustrated in Figure **S11**. The earlier emergence of the O–O vibrational mode in chiral L‐LDH indicates that the CISS effect considerably lowers the energetic barrier for O–O coupling, thereby enhancing OER activity compared to the achiral DL‐LDH counterpart. Chirality‐induced spin selectivity promotes spin‐polarized electron transfer, facilitating the formation of triplet O_2_ and stabilizing key O–O intermediates at lower overpotentials. Figure 4c,d schematically illustrate the proposed mechanism for spin‐selective electron transport during the OER, highlighting the critical difference between the achiral and chiral systems. In the achiral CoFe‐LDH (Figure 4c), the material lacks spin selectivity and does not function as a spin filter. Consequently, electrons with random spin orientations (both spin‐up and spin‐down) are transferred from the reactant (e.g., *OH), resulting in spin‐antiparallel intermediates. This random spin configuration has a higher energy barrier to the formation of triplet O_2_ from these intermediates, resulting in sluggish kinetics. Conversely, the chiral CoFe‐LDH (Figure 4d), enabled by the CISS effect of the intercalated phenylalanine molecules, acts as an effective spin filter. It selectively blocks one spin orientation while preferentially allowing the other (e.g., spin‐down, as depicted) to pass through. This process ensures that the reaction proceeds via spin‐polarized intermediates (*OH) that are in a spin‐parallel (spin‐aligned) configuration, thus facilitating a spin‐allowed pathway for the formation of triplet O_2_ and significantly enhancing the OER activity. Figure 4e,f presents the schematic energy diagrams of achiral and chiral CoFe‐LDH for the OER, where the spin‐polarized intermediates have a lower energy barrier for the OER compared to their spin‐unpolarized counterparts. Through molecular intercalation of chiral Phe molecules into CoFe‐LDH, the resulting CISS effect directs the OER along a lower‐energy reaction pathway. This spin‐selective process substantially reduces the OER energy barrier, resulting in a notable acceleration of reaction kinetics and overall catalytic efficiency. The direct in situ comparisons between achiral DL‐LDH and chiral LDH catalysts establish catalyst chirality and spin selectivity as the dominant factors responsible for the enhanced OER performance.

**FIGURE 5:**
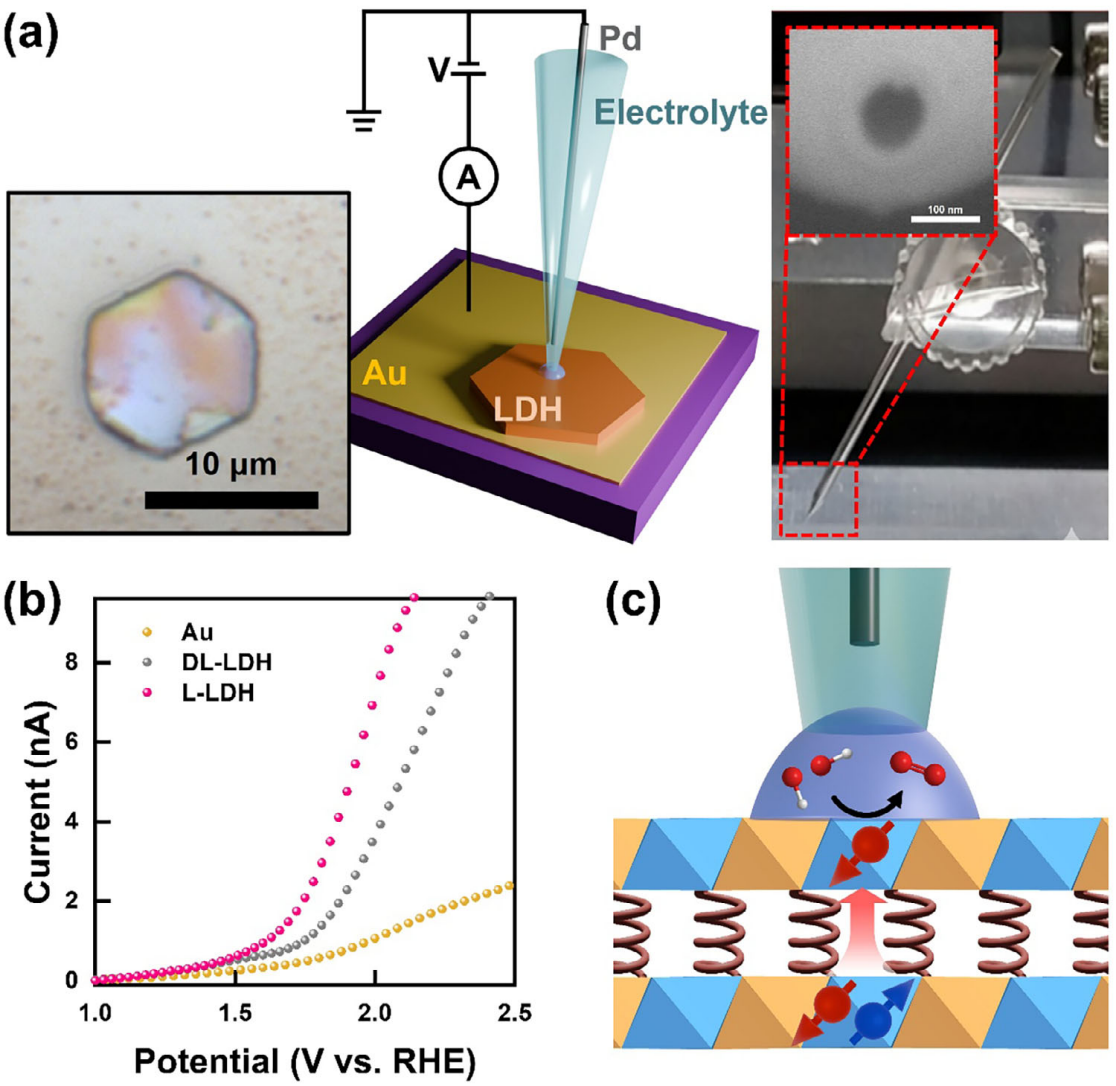
(a) Schematic illustration of the SECCM setup. The optical image (left) shows the LDH microplate deposited on an Au substrate. The SECCM experiments were carried out using a nanopipette probe filled with 50 mM KOH electrolyte. The photograph (right) and the inset SEM image display a nanopipette probe with d_tip_ = 100 nm. (b) LSV curves of individual L‐LDH and DL‐LDH microplates compared to the baseline response obtained on a bare Au substrate. (c) Scheme illustrating the spin filtering effect in chiral LDH microplate during the OER process.

Finally, we employed nanoscale SECCM to probe the chirality‐induced spin‐selective OER on individual Phe‐intercalated CoFe‐LDH flakes, enabling direct insight into local catalytic behavior while eliminating the possible ensemble‐averaging effect. SECCM employs a nanopipette, a localized and mobile electrochemical cell, as a probe to offer spatial surface electrochemical characterizations with a resolution of approximately 100 nm. The details of SECCM measurements are provided in the **SI**. Figure 5a presents a schematic of the SECCM measurement on individual DL‐ and L‐CoFe‐LDH microplates deposited on a Si substrate covered with a thin Au film. A scanning nanopipette containing electrolyte and a palladium (Pd) wire as a quasi‐reference counter electrode (QRCE, 800 mV vs. RHE) was used to probe the catalyst. Figure 5b shows localized LSV curves for DL‐ and L‐CoFe‐LDH catalysts, revealing that the chiral L‐LDH microplate delivers ∼40% higher OER current at 1.6 V vs. RHE compared to the achiral DL‐LDH. Figure 5c highlights the nanoscopic junction formed at the probe–catalyst contact. This directly confirms that the enhanced activity originates from chiral‐induced spin‐polarized OER, where electron transfer follows a spin‐selective pathway enabled by molecular intercalation of chiral molecules and the resulting CISS effect.

## 3 Conclusion

In summary, this work demonstrates a strategy for enhancing oxygen evolution reaction (OER) performance by intercalating chiral phenylalanine molecules into CoFe‐LDH catalysts. This approach not only expands the interlayer spacing, providing more accessible active sites, but—more importantly—introduces spin selectivity via the CISS effect. The intercalated chirality acts as a spin filter, directing the reaction along a lower‐energy pathway that generates triplet‐state O_2_. Chiral molecules such as L‐ and D‐phenylalanine induce preferential spin alignment of charge carriers without the application of an external magnetic field, facilitating spin‐selective charge transfer and promoting spin‐favored O─O bond formation. In situ XANES and complementary spectroscopic analyses confirm that Co ions are the active sites and that chirality accelerates their oxidation, providing direct evidence of chirality‐induced spin‐polarized OER enhancement. These findings establish a new paradigm for the rational design of advanced oxide‐based electrocatalysts, highlighting the critical role of spin dynamics in modulating reaction pathways. Beyond OER, this work suggests that molecular chirality could be a versatile tool for controlling spin‐polarized electron transfer in other electrochemical transformations, paving the way for the development of highly selective and efficient spin‐engineered catalytic systems. Overall, this study provides fundamental insights into the interplay between molecular chirality, spin selectivity, and catalytic activity, offering a promising strategy to optimize energy conversion processes in water splitting and beyond.

## Author Contributions

**Chia‐Che Chang**: methodology, investigation. **Yu‐Chang Lin**: resources, methodology, validation. **Wei‐Tsung Chuang**: resources, methodology. **Che‐Lun Lee**: investigation, methodology. **Jessie Shiue**: resources, methodology, validation. **Cheng‐Rong Wu**: methodology, investigation, validation, data curation. **Huang‐Ming Tsai**: methodology, resources. **Ya‐Lun Ho**: data curation, resources, methodology, validation, investigation. **Yu‐Ying Chang**: investigation, methodology, validation. **Chih‐Ying Huang**: writing – original draft, methodology, investigation, visualization, validation, data curation. **Yang‐Sheng Lu**: methodology, investigation, validation, writing – original draft, data curation, visualization. **Way‐Faung Pong**: resources, supervision, conceptualization, writing – review and editing. **Tsung‐Hsin Liu**: methodology, investigation. **Hua‐Shu Hsu**: resources, validation, methodology. **Chia‐Chun Chen**: resources, supervision. **Jau‐Wern Chiou**: validation, methodology, resources. **Shao‐Sian Li**: resources, methodology. **Chun‐Wei Chen**: supervision, resources, conceptualization, funding acquisition, writing – review and editing.

## Conflicts of Interest

The authors declare no conflicts of interest.

## Supporting information




**Supporting File**: advs76090‐sup‐0001‐SuppMat.docx.

## Data Availability

The data that support the findings of this study are available from the corresponding author upon reasonable request.
