# Supplementary material for: Spin‐Selective Oxygen Evolution in Chiral Molecule‐Intercalated Layered Double Hydroxides
Source: Adv Sci (Weinh). 2026 Jun 16;13(50):e76090. doi: 10.1002/advs.76090 (PMC13336434; doi:10.1002/advs.76090)
Supplement: Supplementary file 1 [file ADVS-13-e76090-s001.docx]

**Supporting Information**

**Spin-Selective Oxygen Evolution in Chiral Molecule-Intercalated Layered Double Hydroxides**

Chih-Ying Huang^1,2^, Cheng-Rong Wu^3^, Yang-Sheng Lu^4^, Yu-Ying Chang^5^, Chia-Che Chang^6^, Tsung-Hsin Liu^1,2^, Che-Lun Lee^3^, Jessie Shiue^7^, Yu-Chang Lin^8^, Wei-Tsung Chuang^8^, Huang-Ming Tsai^8^, Ya-Lun Ho^9^, Jau-Wern Chiou^10^, Hua-Shu Hsu^5^, Shao-Sian Li^4^, Chia-Chun Chen^11^, Way-Faung Pong^12^, Chun-Wei Chen^1,3,13,*^

1. *International Graduate Program of Molecular Science and Technology (NTU-MST), National Taiwan University, Taipei 10617, Taiwan*
2. *Molecular Science and Technology Program Taiwan International Graduate Program (TIGP), Academia Sinica, Taipei 115201, Taiwan*
3. *Department of Materials Science and Engineering, National Taiwan University, Taipei 10617, Taiwan, Email: chunwei@ntu.edu.tw*
4. *Department of Materials Science and Mineral Resources Engineering, National Taipei University of Technology, Taipei 10608, Taiwan*
5. *Department of Applied Physics, National Pingtung University, Pingtung County 900391, Taiwan*
6. *Graduate Institute of Nanomedicine and Medical Engineering, College of Biomedical Engineering and International Ph.D. Program in Biomedical Engineering, College of Biomedical Engineering, Taipei Medical University, Taipei 110301, Taiwan*
7. *Institute of Atomic and Molecular Sciences, Academia Sinica, Taipei 10617, Taiwan*
8. *National Synchrotron Radiation Research Center, Hsinchu 300, Taiwan*
9. *Research Center for Electronic and Optical Materials, National Institute for Materials Science (NIMS), Ibaraki 305-0044, Japan*
10. *Department of Applied Physics, National University of Kaohsiung, Kaohsiung 811, Taiwan*
11. *Department of Chemistry, National Taiwan Normal University, Taipei 116, Taiwan*
12. *Department of Physics, Tamkang University, New Taipei 251301, Taiwan*
13. *Center for Condensed Matter Sciences and Center of Atomic Initiative for New Materials (AI-MAT), National Taiwan University (NTU), Taipei 10617, Taiwan*

**Material synthesis**

The pristine CoFe-LDH was prepared using a co-precipitation method. Solution A was prepared by dissolving 45 mmol of Co(NO_3_)_2_·6H_2_O and 15 mmol of Fe(NO_3_)_3_·9H_2_O in 50 mL of deionized water, forming a homogeneous solution with a Co:Fe molar ratio of 3:1. Solution B was prepared by dissolving 40 mmol of Na_2_CO_3_ and 96 mmol of NaOH in 25 mL of deionized water. Solutions A and B were then mixed together, and the pH was adjusted to 8.5 using 1 M NaOH. The mixture was transferred into a round-bottom flask for the reaction. A thermocouple was used to precisely control the heating mantle temperature, heating the mixture to 80 ℃ while stirring with a magnetic stir bar for 18 hours. After the reaction was completed, the product was washed three times with isopropyl alcohol. Finally, centrifugation was performed at 5000 rpm for 15 minutes, the supernatant was discarded, and the precipitate was collected.

The synthesis method of chiral CoFe-LDH is similar to that of the pristine CoFe-LDH, also prepared via a co-precipitation method. Solution A was prepared by dissolving 1.2 mmol of Co(NO_3_)_2_·6H_2_O and 0.4 mmol of Fe(NO_3_)_3_·9H_2_O in 16 mL of deionized water, forming a homogeneous solution with a Co:Fe molar ratio of 3:1. Solution B was prepared by dissolving 1.6 mmol of DL-, L-, and D-phenylalanine (DL-, L-, and D-Phe) chiral molecules in 80 mL of deionized water. Solutions A and B were then mixed together, and the pH was adjusted to 8.5 using 1 M NaOH. The mixture was transferred into a round-bottom flask for the reaction. A thermocouple was used to precisely control the temperature of the heating mantle, heating the mixture to 80 ℃ while stirring for 3 hours. Unlike the pristine CoFe-LDH synthesis, in order to successfully incorporate the chiral molecules, it is necessary to avoid contamination from atmospheric CO_2_. Therefore, the entire reaction process was conducted under an inert gas atmosphere. After the reaction was completed, the product was washed three times with isopropyl alcohol. Finally, centrifugation was performed at 5000 rpm for 15 minutes, the supernatant was discarded, and the precipitate was collected.

**Materials characterization**

The TEM images were performed in FEI Tecnai T12. The SEM images were collected through NOVA NANO SEM 450. The CD spectra were measured by JASCO J-815. The powder X-ray diffraction patterns were measured by using Bruker D8 with Cu *K*_α_ radiation (λ = 1.5418 Å). Raman spectra were performed by a homemade system equipped with continuous Nd:YAG 532 nm laser, Olympus microscope (50X objective lens), Andor Kymera 193i-B2 spectrometer, and Andor iDus416 low-noise detector. TGA analysis were measured by SDT Q600 V20.9 Build 20.

**Electrochemical characterization**

The OER reaction were operated in a three-electrode system with a working electrode of Ni foam, a counter electrode of Pt wire, and an Hg/HgO (1 M KOH) reference electrode. The catalyst electrode was fabricated using the drop-casting method. The LDH catalysts were dispersed in IPA/H_2_O (v/v =1:4) solvent, followed by adding Naﬁon as the binder. The mixtures were ultrasonicated for 10 min to reach a homogeneous solution. Finally, the as-prepared solution (500 μL) was dropped onto Ni foam electrodes and dried at room temperature. Linear sweep voltammetry (LSV) curves were performed in O_2_-saturated 1 M KOH_(aq)_ by using Autolab PGSTAT204 with the FRA32M EIS module. The LSV curves were recorded with a scan rate of 5 mV s^-1^ in a potential range of 1.2 to 1.8 V vs. RHE. Electrochemical impedance spectroscopy (EIS) was performed at frequencies between 10 kHz and 0.01 Hz with an overpotential of 270 mV (about 1.5 V vs. RHE). All potentials were converted to the RHE scale according to the following equation: E_RHE_ = E_Hg/HgO_ + 0.098 V + 0.059 V × pH with 90% iR correction.

**Scanning electrochemical cell microscopy (SECCM) measurement**

We employed SECCM to assess the catalytic performance of Phe-intercalated CoFe-LDH flakes in OER. A Pd wire with a diameter of approximately 0.25 mm was inserted into borosilicate about 100 nm in diameter, filled with electrolytes, serving as a quasireference counter electrode (QRCE). The potential of the chiral CoFe-LDH (as a working electrode) was controlled relative to the Pd QRCE at the tip, while the current was monitored with a variable-gain transimpedance amplifier. In this work, the LSV scan rate was set at 100 mV s^-1^, and the measurement time was 20 ms per point.

**Synchrotron radiation-based spectroscopic measurement**

The in situ Co and Fe *K*-edge XANES were performed at the Taiwan Light Source (TLS) 13B2 beamline (NSRRC, Taiwan). Data acquisition was carried out in fluorescence mode. The energy positions were concurrently calibrated against metallic Co and Fe foil standards. Electrochemical conditions were controlled within a specialized Teflon-based cell, featuring a standard three-electrode setup submerged in a 1 M KOH alkaline medium.

The ex situ Fe and Co *L*_3,2_-edge XANES were measured in total electron yield mode at room temperature using Taiwan Photon Source (TPS) 45A2-undular beamline of NSRRC. The samples were measured in an ultrahigh vacuum chamber. The angle of incidence of the incoming photon with respect to the sample surface was 45°. The resolution was set to 0.1 eV at a 700-800 eV photon energy during XANES measurement. The spectra were normalized using the incident beam intensity, I_0_, and keeping the equal area under the spectra in the energy range 730-740 eV and 805-815 eV, respectively.

**Supplementary Figures**

**Figure S1.** TEM images of (a) pristine, (b) DL-, (c) D-, and (d) L-LDH.

**Figure S2.** AFM images of (a) pristine, (b) DL-, (c) D-, and (d) L-LDH.

**Figure S3.** AFM height profile of (a) pristine, (b) DL-, (c) D-, (d) L-LDH.

**Figure S4.** EDS mapping of (a) pristine, (b) DL-, (c) D-, and (d) L-LDH.

**Figure S5.** CD spectra of DL-, D-, and L-Phe molecules solution (1 mM).

**Figure S6.** Cyclic voltammetry (CV) curves of (a) pristine, (b) DL-, (c) L-, and (d) D-LDH catalyzed OER collected at various scan rates from 5 to 45 mV s^-1^.

**Figure S7.** The schematic of the spin electron transferring in achiral and chiral CoFe-LDH.

**Figure S8.** (a) Illustration and (b) image of in situ XANES setup.

**Figure S9.** (a) Fe and (b) Co L_3,2_-edge XANES spectra of DL- and L-LDH together with CoO and Fe_2_O_3_.

**Figure S10.** (a) in situ Raman spectra of DL-LDH and (b) L-LDH.

**Figure S11.** Illustration of active intermediate in lattice oxygen mechanism of CoFe-LDH during the OER process.

**Table S1.** Comparison of the OER performance of chiral molecular-coated and intercalated.

| **Catalysts** | **Chiral molecules** | **Overpotential** | **Tafel slope** | **Substrate** | **Electrolyte** | **Reference** |
| --- | --- | --- | --- | --- | --- | --- |
| Fe_3_O_4_ | (R)-1,2-diaminopropane dihydrochloride | 620 mV at 1 mA cm^-2^ | - | glassy carbon | 1 M KOH | Nano Lett. 23, 19, 9042–9049 (2023) |
| NiFe-LDH | R-Perylene diimide polymer | 254 mV at 10 mA cm^-2^ | - | FTO | 1 M KOH | J. Mater. Chem. A, 12, 20354–20363 (2024) |
| Co@CoO | L-Cysteine | 300 mV at 10 mA cm^-2^ | 84.2 mV dec^-1^ | Pt | 1 M NaOH | Chem. Eng. J. 493: 152545 (2024) |
| 23% Fe-doped Co_3_O_4_ | L-Cysteine | 320 mV at 10 mA cm^-2^ | 34 mV dec^-1^ | glassy carbon | 1 M NaOH | Nat Commun 14, 1067 (2023) |
| L-Ni:1T-MoS_2_ | L-Ascorbic acid | 320 mV at 10 mA cm^-2^ | 60 mV dec^-1^ | glassy carbon | 1 M KOH | J. Power Sources, 641, 236839 (2025) |
| CoFe-LDH | D-Phenylalanine | 245 mV at 10 mA cm^-2^ | 69.1 mV dec^-1^ | Ni foam | 1 M KOH | This work |
| CoFe-LDH | L-Phenylalanine | 245 mV at 10 mA cm^-2^ | 67.8 mV dec^-1^ | Ni foam | 1 M KOH | This work |
